# Potential of flow injection analysis for dissolution tests: application to on-line monitoring of tablets with different dissolution rates

**DOI:** 10.1155/S1463924690000189

**Published:** 1990

**Authors:** J. S. Cosano, A. Izquierdo, M. D. Luque de Castro, M. Valcárcel

**Affiliations:** Department of Analytical Chemistry Faculty of Sciences University of Córdoba Córdoba Spain


Journal of Automatic Chemistry, Vol. 12, No. 4 (July–August 1990), pp. 145–148

### Potential of flow injection analysis for dissolution tests: application to on-line monitoring of tablets with different dissolution rates

**J. S. Cosano, A. Izquierdo, M. D. Luque de Castro and M. Valcárcel**

*Department of Analytical Chemistry, Faculty of Sciences, University of Córdoba, Córdoba, Spain*

*The possibilities of flow injection analysis for the on-line monitoring of dissolution tests are illustrated by using a very straightforward manifold to monitor tablets of ascorbic acid dissolving at different rates. The dissolution kinetics can be monitored at an adequate sampling frequency as the method allows up to 90 samples to be processed per hour.*

Flow Injection Analysis (FIA) has proved to be a useful tool for on-line process monitoring [1,2] and pharmaceutical analysis [3–5]. Both applications are of great importance in the monitoring of dissolution tests of solid dosage forms. Dissolution tests on pharmaceuticals are commonplace quality control procedures and facilitate meeting the legal requirements for compendial drugs, and for oral-dosage forms which are not yet listed in compendia.

Conventional approaches to the monitoring of dissolution processes occurring simultaneously in the 'controller', according to the requirements established by pharmacopoeia regulations, include the following: (1) an automated selecting valve sequentially driving a small volume of the evolving system to the single flow-cell of a photometric detector, or to a dedicated detector with six flow-cells, each receiving the stream from one vessel (both alternatives monitoring the native absorbance of an active compound); (2) the use of a sampler as a interface between the dissolution vessel and the instrument (generally a high-pressure liquid chromatograph); and (3) in-line monitoring. These approaches suffer from a series of shortcomings such as: (*a*) the difficulty of controlling non-specific, or non-selective, active compounds (for example the case of absorption spectra overlapping with those of other components in the oral-dosage); (*b*) the process is controlled in anything but real-time – very expensive instrumentation and maintenance costs would be involved if real-time was used; (*c*) measurement errors arise from the presence of variable amounts of suspended solids and these result in complex and imprecise data treatment.

The need for new instrumentation and methods for monitoring of dissolution tests increases day by day, fostered by the manufacture of new drugs with widely different dissolution rates, and by the need for stricter quality control as a result of the growing competition in international markets.

This paper aims to show the suitability of FIA in adapting existing methodologies to the kinetics of the dissolution process. A photometric method was developed for the determination of ascorbic acid, and then adapted to the monitoring of this analyte in tablets with fast and slow dissolution kinetics.

### Experimental

### *Instruments and apparatus*

A Perkin-Elmer Lambda-1 single-beam spectrophotometer was used. This was equipped with a Hellma 178 12QS flow-cell and connected to a Radiometer REC-80 recorder, and Hewlett-Packard PH-85 microcomputer, through an HP-IB 82937A interface. A Gilson Minipuls-2 four-channel peristaltic pump, a Tecator L-100 injection valve and two Omnifit 2407 confluence points were also used.

### *Reagents*

Aqueous solutions of $3 \times 10^{-4}$ M chloramine T, 0.2 M $H_2SO_4$, starch potassium iodide mixture (1 g/l soluble starch + 2 g/l KI) and standards of ascorbic acid were prepared daily.

### Results and discussion

### *Manifold and chemical system*

The manifold used for the optimization of variables is shown in figure 1; the dissolution vessel was replaced with a flask containing the sample solution of ascorbic acid. This sample, dissolved in an acidic medium, was injected into the chloramine T-stream after merging with a starch-iodide stream. Iodide forms HI in an acid media, which subsequently reacts with chloramine T. The iodine formed oxidizes vitamin C. Once vitamin C has been fully consumed, iodine binds to starch, which constitutes the indicator reaction. Sulphuric acid is added directly to the injected sample volume. This stream then converges with the chloramine T-stream, thus avoiding precipitation of the reagent. No iodine was consumed when the sample contained only sulphuric acid, due to the absence of vitamin C, and the signal obtained was maximum. The presence of vitamin C reduced the analytical signal in proportion to its concentration.

0142–0453/90 $3.00 © 1990 Taylor & Francis Ltd.

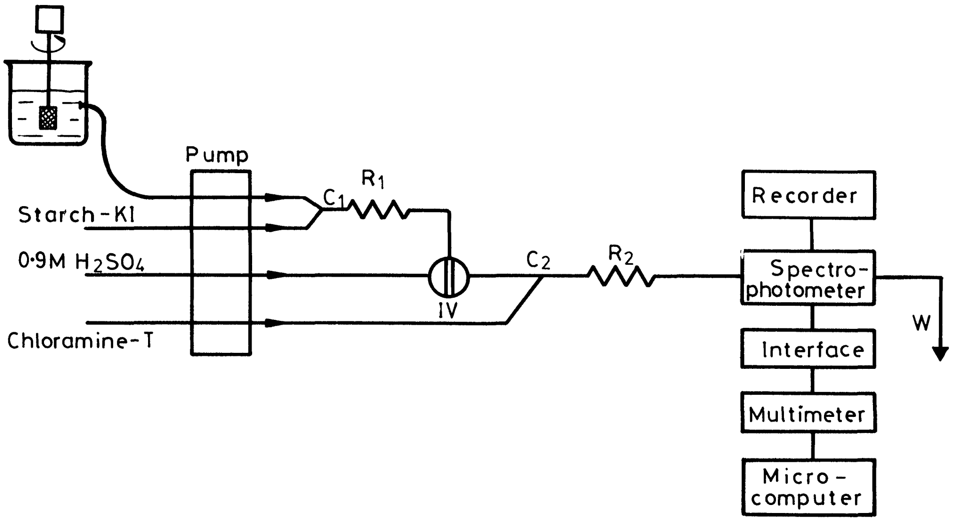

*Figure 1. FIA manifold for the monitoring of dissolution tests on pharmaceutical dosages containing ascorbic acid.*

*Optimization of variables*

The optimum values for the FIA variables (see figure 1) were as follows: flow-rate ($q$) = 2·04 ml/min; injected volume ($V_i$) = 96·2 µl; reactor length ($R_1$, $R_2$) = 50 and 55 cm, respectively; inner diameter of the reactors ($\phi$) = 0·5 mm. These values allowed the blue colour of the starch–iodine complex to be formed as the sample plug passed through the detector, then it was monitored at 581 nm. The optimization of the variables ensured the maximum difference between the reference and the sample peaks.

The influence of the chloramine T concentration was one of the most important variables in this determination. At high concentrations of chloramine T, the presence of vitamin C had no effect on the analytical signal. The maximum difference between the blank and the sample signal was obtained with $3 \times 10^{-4}$ M chloramine T solutions. At lower concentrations, the absorbance difference decreased owing to the drastic weakening of the blank signal.

The pH of the sample was adjusted by adding $H_2SO_4$. The reaction medium must be acidic to allow the formation of HI. The absorbance difference between the blank and the sample was high at low pH. At sulphuric acid concentrations above 0·75 M the signal difference increased only slightly and some peak broadening was observed. A 0·90 M sulphuric acid concentration was chosen as optimum. The most suitable concentrations of KI and starch are set out in the 'Experimental' section of this paper.

It is well known that high temperatures hinder the development of the blue colour of the starch-iodine complex; therefore, the experiments were performed at room temperature.

*Determination of vitamin C*

This method allows the determination of vitamin C from the equation:

$$A = 0{\cdot}117 + 0{\cdot}003\ [\text{vitamin C}];\ r = 0{\cdot}999$$

where $A$ is the absorbance difference between the blank and the sample containing vitamin C (whose concentration is expressed in µg/ml). The equation covers the calibration range between 15 and 150 µg/ml, the r.s.d. ($p = 0{\cdot}05$) for the determination of 100 µg/ml of vitamin C being 0·97%. The sampling frequency was 90 $h^{-1}$.

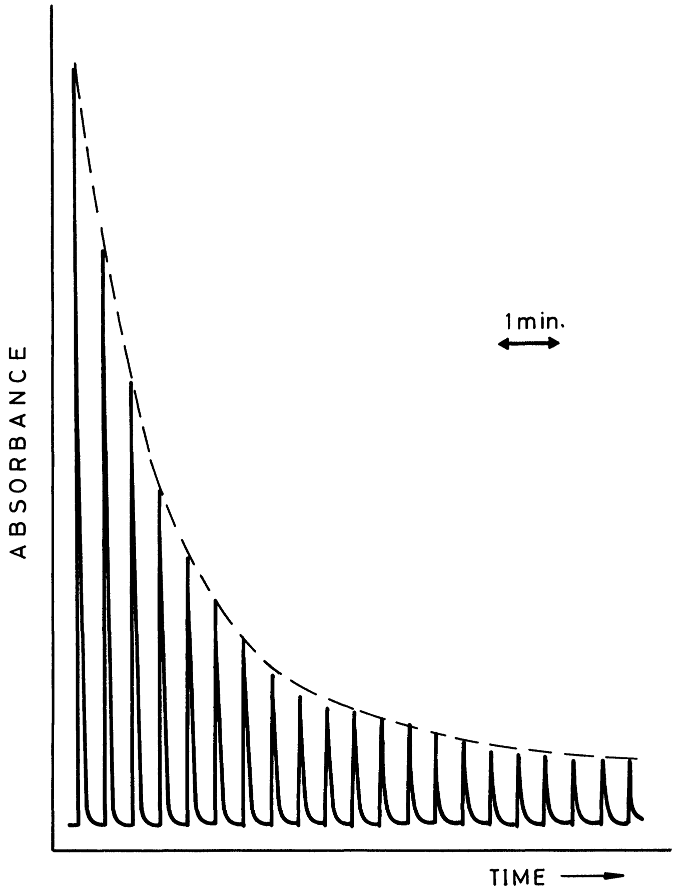

*Figure 2. Recording obtained in the monitoring of tablets with fast dissolution kinetics. The envelope of the maxima of the peaks shows the nature of the kinetics.*

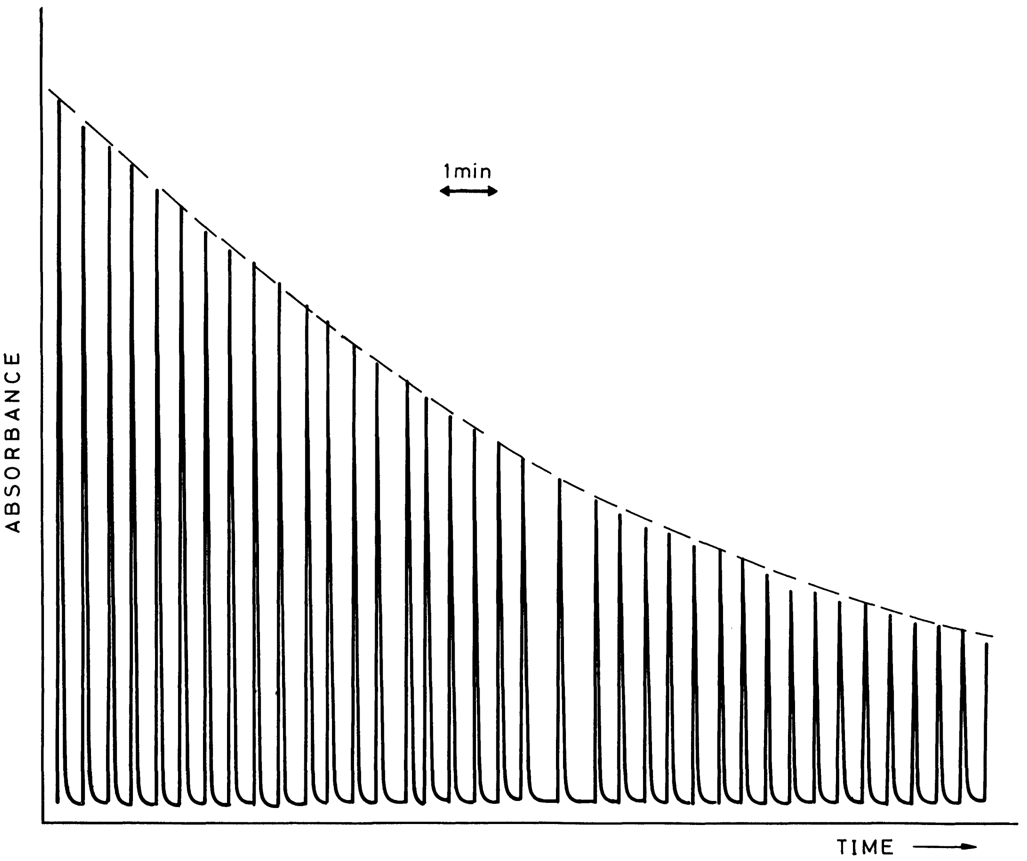

*Figure 3. Recording obtained in the monitoring of tablets of slow dissolution kinetics. The envelope is representative of the dissolution rate.*

*On-line monitoring of ascorbic acid dosage forms with different dissolution kinetics*

Prior to starting the dissolution process, a triplicate injection of the dissolving solution was performed and the average of this blank signal was stored in the microcomputer. Then, the tablet was introduced into the stirring basket and injection from the solution in the vessel were performed at preselected time intervals, the absorbance-time data were also collected by the microcomputer (the blank value and the calibration curve had been previously input). In this way, a simple program written in BASIC was used to collect a table of time–analyte concentration values and, hence, the concentration of ascorbic acid at any moment during the process. A recorder connected to the detector also allows the visual monitoring of the process through the recording of the peaks corresponding to the evolution of the tablet. The envelope of the maxima of the FIA peaks was a kinetic curve of the dissolution process. The approach was tested with two types of ascorbic acid dosage forms, Treasury and Redoxon, with different dissolution kinetics because Treasury (timed release tablets) were furnished with a special molecular sieve which retarded the dissolution process, while Redoxon tablets have a very fast dissolution rate. Figures 2 and 3 show the recordings for the two tablets; the fast dissolution of the Redoxon tablets (figure 2) and the slower rate of the Treasury tablets (figure 3) are very clear.

### Final remarks

This paper shows the value of FIA for on-line monitoring of dissolution tests. The method developed here features a number of interesting advantages:

(1) Small sample consumption (the sample can be returned to the vessel once the loop of the injection valve has been filled).

(2) A derivatizing reaction can be readily implemented to improve analytical features of the determination, such as sensitivity and/or selectivity. In addition, reagent consumption is very small and a variety of chemical systems (as described by Koupparis *et al.* [6–9]) and detectors can be used.

(3) The response is obtained in near to real-time, in contrast with the conventional devices used for this purpose.

(4) The high sample throughput allows frequent monitoring as required, by the rate of real-time being studied. This is of great value for fast dissolution kinetics.

(5) In addition, the on-line separation technique can be readily implemented whenever the analytes require such treatment [10].

(6) FIA can be adapted to the monitoring of any type of dissolution kinetics demonstrated in this work.

### Acknowledgement

The authors would like to thank CICyT for financial support (Grant No. PA86-0146).

### References

1. van der Linden, W.R., *Analytica Chimica Acta,* **179** (1986), 91.
2. Luque de Castro, M. D., *Talanta*, **36** (1989), 591.
3. Ma, G. and Jiang, D., **8** (1988), 248.
4. Ríos, A., Luque de Castro, M. D. and Valcárcel, M., *J. Pharm. Biomed. Anal.*, **3** (1985), 105.
5. Luque de Castro, M. D. and Valcárcel, M., *J. Pharm. Biomed. Anal.* (in press).
6. Koupparis, M., Macheras, P. and Reppas, C., *International Journal of Pharmaceutics*, **20** (1984), 325.
7. Koupparis, M., Macheras, P. and Tsaprounis, C., *International Journal of Pharmaceutics*, **27** (1985), 349.
8. Koupparis, M. and Marcuchova, A., *Analyst*, **111** (1986), 313.
9. Koupparis, M. and Sarantonis, E. G., *Journal of Pharmaceutical Sciences*, **75** (1986), 800.
10. Nord, L., Sundaren, M. and Torstensson, A., *J. Pharm. Biomed. Anal.*, **7** (1989), 421.

